# A Putative Role for TRPC6 in Immune-Mediated Kidney Injury

**DOI:** 10.3390/ijms242216419

**Published:** 2023-11-16

**Authors:** Daan C. ‘t Hart, Johan van der Vlag, Tom Nijenhuis

**Affiliations:** Department of Nephrology, Radboud Research Institute for Medical Innovation, Radboud University Medical Centre, 6500 HB Nijmegen, The Netherlands; daan.thart@radboudumc.nl (D.C.‘t.H.); johan.vandervlag@radboudumc.nl (J.v.d.V.)

**Keywords:** TRPC6, immune-mediated kidney injury, calcium, podocyte

## Abstract

Excessive activation of the immune system is the cause of a wide variety of renal diseases. However, the pathogenic mechanisms underlying the aberrant activation of the immune system in the kidneys often remain unknown. TRPC6, a member of the Ca^2+^-permeant family of TRPC channels, is important in glomerular epithelial cells or podocytes for the process of glomerular filtration. In addition, TRPC6 plays a crucial role in the development of kidney injuries by inducing podocyte injury. However, an increasing number of studies suggest that TRPC6 is also responsible for tightly regulating the immune cell functions. It remains elusive whether the role of TRPC6 in the immune system and the pathogenesis of renal inflammation are intertwined. In this review, we present an overview of the current knowledge of how TRPC6 coordinates the immune cell functions and propose the hypothesis that TRPC6 might play a pivotal role in the development of kidney injury via its role in the immune system.

## 1. Introduction to the TRPC6 Channel Family

**Figure 1:**
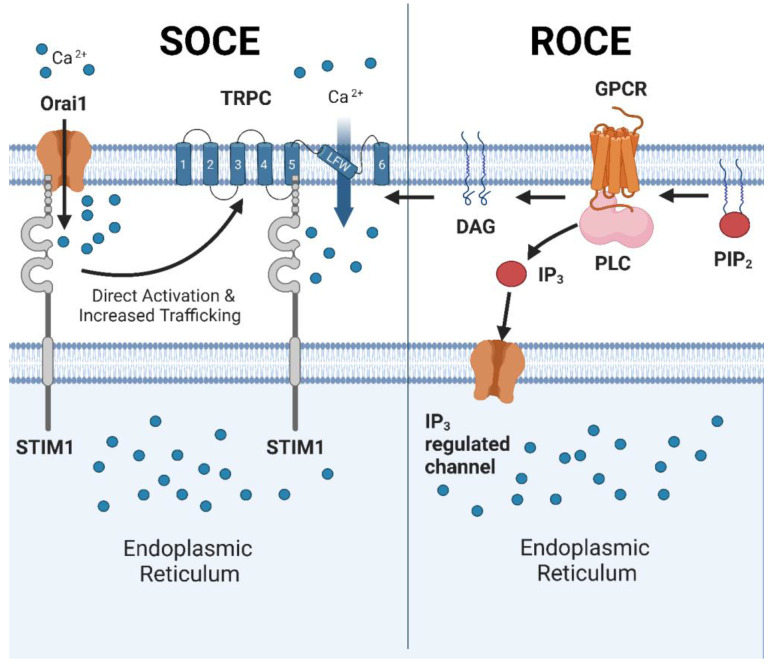
Overview of the activating mechanisms of the TRPC channels. Left panel: store-operated Ca^2+^ entry (SOCE). Depletion of Ca^2+^ in the endoplasmic reticulum (ER) is sensed by stromal interaction protein 1 (STIM-1), which subsequently activates calcium-release-activated calcium channel protein 1 (Orai1). Increased Ca^2+^ influx via Orai1 leads to the direct activation of the TRPC channels or the enhanced trafficking of the TRPC channels to the plasma membrane. For clarity reasons, only one TRPC subunit is depicted in this figure. Upon insertion in the plasma membrane, the TRPC channel activity is tightly regulated via an interaction with STIM1. Right panel: receptor-operated Ca^2+^ entry (ROCE): G-protein-coupled receptor (GPCR) activation leads to the activation of phospholipase C (PLC), which converts phosphatidylinositol 4,5-bisphosphate (PIP2) to diacyl glycerol (DAG) and inositol 1,4,5-trisphosphate (IP3). IP3 interacts with the IP3 receptor (IP3R) leading to Ca^2+^ release from the ER. DAG is known to directly stimulate the activity of TRPC3, TRPC6 and TRPC7. Abbreviations: diacyl glycerol (DAG); endoplasmic reticulum (ER); inositol 1,4,5-trisphosphate (IP3); g-protein-coupled receptor (GPCR); IP3 receptor (IP3R); calcium-release-activated calcium channel protein 1 (Orai1); phospholipase C (PLC); phosphatidylinositol 4,5-bisphosphate (PIP2); receptor-operated Ca^2+^ entry (ROCE); store-operated Ca^2+^ entry (SOCE); stromal interaction protein 1 (STIM-1). Image created using Biorender.com.

The transient receptor potential channel C (TRPC) superfamily includes seven closely related cation channels with a high permeability for Ca^2+^ [1]. Based on their structural homology, the TRPC family can be subdivided into two subfamilies, of which TRPC1, TRPC2, TRPC3, TRPC6 and TRPC7 form one subfamily, and TRPC4 and TRPC5 form the other subfamily [2]. TRPC3, TRPC6 and TRPC7 are very closely related with ~75% amino acid homology [3], whereas TRPC2 is known as a non-functional pseudogene in humans [4]. All of the TRPC family members share a common subunit structure consisting of a cytoplasmic C- and N-terminus and six transmembrane domains (Figure 1) [2]. Between transmembrane domains 5 and 6, a putative pore is formed, thereby enabling the transport of Ca^2+^ and other ions across the plasma membrane. To create a functional TRPC channel, four subunits assemble in a monomeric or heteromeric fashion [5,6,7]. 

TRPC membrane activity is, amongst others, regulated by controlled trafficking of the TRPC channels to the plasma membrane, e.g., via calmodulin activation [8,9]. The membrane-inserted TRPC channels are constitutively active or need to be activated via either the receptor-operated Ca^2+^ entry (ROCE) pathway or the store-operated Ca^2+^ entry (SOCE) pathway [8,9,10]. During ROCE, activation of a G-protein-coupled receptor (GPCR) leads to the activation of phospholipase C (PLC) [11,12,13]. PLC catalyzes the conversion of phosphatidylinositol 4,5-bisphosphate (PIP2) to diacyl glycerol (DAG) and inositol 1,4,5-trisphosphate (IP3). DAG directly activates TRPC3, TRPC6 and TRPC7 [11,12]. This still remains controversial for the other TRPC family members [14]. 

During SOCE, depletion of the Ca^2+^ levels in the endoplasmic reticulum (ER) leads to the activation of the ER-resident protein stromal interaction protein 1 (STIM-1) [15]. STIM1 stimulates calcium-release-activated calcium channel protein 1 (Orai1), resulting in a Ca^2+^ influx into the cell. The Orai1-mediated Ca^2+^ gradients lead to both the direct activation of TRPC channels as well as the increased trafficking and insertion of the TRPC channels into the plasma membrane. The gating of the TRPC channels upon insertion in the plasma membrane is tightly regulated by STIM1 [15]. The activities of TRPC1, TRPC3 and TRPC4 are the best characterized as being regulated by STIM-1-Orai1, whereas TRPC6 activity is primarily known to be activated by DAG [15,16].

## 2. Physiological Function of TRPC6

The TRPC family exerts a wide array of (patho)physiological functions. In this review we will focus on the (patho)physiological role of TRPC6. TRPC6 is well-known to be expressed in tissues containing smooth muscle cells, e.g., the lungs, the oesophagus, the colon, the vasculature and the stomach [17]. Studies have shown that TRPC6 tightly regulates the Ca^2+^ currents in smooth muscle cells and consequently smooth muscle cells tone. TRPC6 is therefore an important factor influencing blood pressure, intestinal motility and hypoxic pulmonary vasoconstriction [17,18,19,20]. TRPC6 has also been shown to be expressed in the heart, where it regulates Ca^2+^ currents and cardiac function [21,22]. A detailed description of all physiological functions of TRPC6 is beyond the scope of the current paper, and we kindly refer additional inquiries to the recent review by Dietrich et al. [17]. In this review, we will focus on the role of TRPC6 in the kidneys and the immune system with particular emphasis on how the function of TRPC6 in regulating the immune response is linked to the pathogenesis of immune-related kidney diseases. 

## 3. TRPC6 in the Kidneys

**Figure 2:**
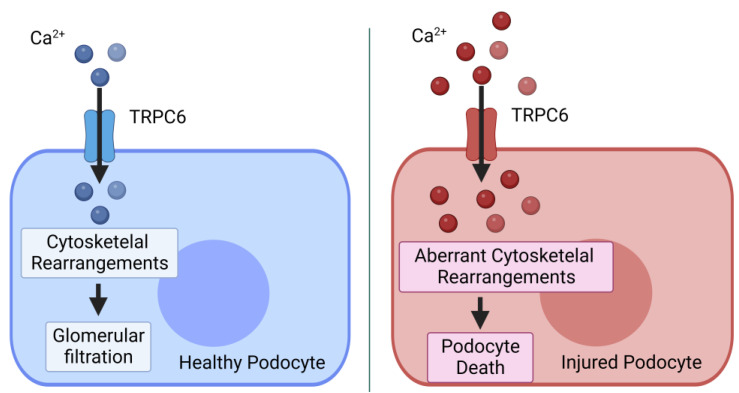
The non-immunological roles of TRPC6 in the podocyte. Left panel: Under physiological conditions, Ca^2+^-transients via TRPC6 are required for cytoskeletal rearrangements. These cytoskeletal rearrangements are pivotal in responding to alterations in mechanical forces, like altered blood pressure, and consequently for preserving glomerular filtration. Right panel: Pathological conditions, e.g., a gain-of-function mutation in TRPC6, result in an increased Ca^2+^ influx via TRPC6 into podocytes. The increased Ca^2+^ influx leads to aberrant cytoskeletal rearrangement and podocyte injury and/or death. Image created using Biorender.com.

The kidneys are the organs responsible for maintaining the homeostasis of the body’s internal milieu by excreting toxic substances and by regulating salt and water balance. The functional unit of the kidney is the nephron, consisting of the glomerulus, the proximal tubule, Henle’s loop, the distal tubule and the collecting duct. TRPC6 is known to be expressed in the collecting duct as well as in all three cell types of the glomerulus (i.e., podocytes, glomerular endothelial cells and mesangial cells) [23,24,25,26]. The role of TRPC6 is primarily characterized in glomerular podocytes [26]. However, this does not exclude the possibility that TRPC6 plays an important role in other glomerular cell types, i.e., glomerular endothelial cells or mesangial cells [26,27]. Podocytes are a crucial component of the glomerular filtration barrier and restrict the passage of proteins from blood to the urine, mainly in a size-selective manner. TRPC6 is located in the podocyte foot processes at or near the site of the slit diaphragm. The slit diaphragm is a mechanosensitive protein complex that connects the foot processes of adjacent podocytes [26]. The slit diaphragm responds to alterations in mechanical forces, such as altered blood pressure, by dynamically rearranging the podocyte actin cytoskeleton. An intact podocyte cytoskeleton is crucial for glomerular filtration (Figure 2) [28,29,30,31,32]. TRPC6 Ca^2+^ gradients are crucial for the podocyte’s cytoskeletal rearrangement under pathological conditions, but TRPC6 also has a pivotal physiological role in maintaining glomerular integrity under physiological conditions [26]. 

Gain-of-function mutations in TRPC6 lead to familial forms of glomerular disease known as focal segmental glomerulosclerosis (FSGS) [33]. FSGS is characterized by proteinuria, loss of renal function and eventually kidney failure [34]. Gain-of-function mutations in TRPC6 have been shown to lead to both increased channel activity and enhanced trafficking of the channel to the plasma membrane [33]. The increased expression and/or activity of TRPC6 leads to aberrant cytoskeletal rearrangements in podocytes, podocyte foot process effacement and eventually podocyte death (Figure 2) [35,36,37,38]. Loss of podocytes will result in the development of proteinuria, which is decreased filtration capacity of the kidney, and kidney failure. 

Most kidney diseases are not caused by genetic defects, like hereditary forms of FSGS, but are a result of systemic pathogenic mechanisms, e.g., hypertension, diabetes mellitus and auto-immunity. TRPC6-mediated signalling has been shown to be involved in the pathogenesis of these acquired forms of kidney diseases as well. For example, TRPC6 is functionally involved in the progression of diabetic kidney disease (DKD) and acquired forms of FSGS by inducing podocyte injury, podocyte foot process effacement and podocyte loss [39,40,41]. Intriguingly, an increasing number of studies suggests that TRPC6 is also tightly linked to the development of immunological kidney disorders. For example, TRPC6 is strongly associated with the infiltration of immune cells into the kidney and consequently with kidney inflammation in the context of ischemia/reperfusion kidney injury (I/R), unilateral ureteral obstruction (UUO) and DKD [42,43,44,45,46]. These findings suggest that TRPC6 might play an as-yet underappreciated role in the immune system and consequently an important role in the pathogenesis of inflammatory kidney diseases. Taken together, TRPC6 is well known for playing a crucial role in both kidney physiology and pathophysiology at the site of the podocyte by regulating podocyte cytoskeletal integrity. An increasing number of studies suggests, however, that TRPC6 is also a key factor in immune-mediated kidney diseases.

## 4. TRPC6 in Immune Cells

### 4.1. Neutrophils

Neutrophils are part of the first line of defence of the host immune system. An important tool of neutrophils to exert their anti-pathogenic function is the production of reactive oxygen species (ROS) via activation of the NADPH oxidase complex (NOX) [47]. Interestingly, TRPC6 has been shown to be required for the complete activation of NOX and the subsequent ROS production in neutrophils [48]. ROS production is also functionally linked to TRPC6 expression and activity in podocytes [49,50], highlighting the parallel functions of TRPC6 in immune cells and podocytes. TRPC6 also plays an important role in neutrophil migration and chemotaxis in response to the chemo attractants macrophage inflammatory protein-2 (MIP-2) and chemokine ligand 2 (CXCL2) [51,52]. TRPC6 was shown to be crucial for neutrophilic actin remodelling upon stimulation with MIP-2 and CXCL2. TRPC6 knockout resulted in an impaired neutrophil migration in vitro and a defective chemotaxis of neutrophils in vivo. As discussed previously, TRPC6 has been shown to have a similar function in the kidneys by reorganizing the cytoskeleton of podocytes [35,36,37,38]. Interestingly, TRPC6 knockout did not affect the migration of neutrophils in response to N-Formylmethionyl-leucyl-phenylalanine (fMLP) [53]. This finding highlights the context-dependent role of TRPC6 in reassembling the neutrophilic cytoskeleton, as TRPC6 does play a role in MIP-2 and CXCL2-mediated chemotaxis but not in fMLP-mediated chemotaxis. Neutrophilic TRPC6 is also important for the adhesion of neutrophils; TRPC6 increased the β2-integrin activation in neutrophils and the subsequent enhanced ICAM-1 binding in response to chemokine ligand 1 (CXCL1) stimulation [45].

Another important antimicrobial defence mechanism of neutrophils is the formation of neutrophil extracellular traps (NETs). NETs are released from neutrophils and are web-like structures of chromatin decorated with anti-microbial proteins, which serve to immobilize and subsequently kill pathogens [54]. NETs were originally thought to be mainly formed via the canonical NOX-dependent pathway [55]. In the NOX-dependent pathway, ROS, produced via the NOX complex, initiate a cascade of events, eventually resulting in the release of NETs [56,57]. As TRPC6 is known to be important for the complete activation of the NOX in neutrophils and subsequent ROS production, this might suggest that TRPC6 plays a role in the NOX-dependent NET formation [48].

Significantly, an increasing number of studies suggest that NETs can also be formed via a NOX-independent pathway [58,59]. In the NOX-independent pathway, ROS production is not involved in NET formation, and neutrophils release the NETs via nuclear membrane blebbing [60,61,62]. Also of note, NET formation via the NOX-independent pathway is induced by Ca^2+^-ionophores, like A23187, thereby suggesting the important role that the Ca^2+^ transients play in NET formation [63,64,65,66,67,68]. Moreover, the neutrophils from cystic fibrosis (CF) patients are characterized by a disrupted Ca^2+^ homeostasis and subsequently impaired NET formation [69]. The TRPC6 blocker 2-aminoethoxydiphenylborane (2-APB) normalized NET formation and restored the antimicrobial killing capacity of neutrophils from CF patients [69,70]. Taken together, these findings might suggest an important role for TRPC6in NET formation via the NOX- independent pathway by mediating the Ca^2+^ gradients in neutrophils. Moreover, TRPC6 might also be involved in NET formation via the NOX-dependent pathway via the production of ROS (Table 1). The roles of TRPC6 and NETs in immune-mediated kidney injury will be discussed in section seven.

### 4.2. Mast Cells, Macrophages and T-Cells

TRPC6 has also been shown to play a functional role in mast cells, macrophages and T-cells. For example, the TRPC6-mediated Ca^2+^ transients are important for mast cell degranulation [72]. Furthermore, TRPC6 is linked to the phagosoma degradation of pathogens by macrophages. The increased insertion of TRPC6 into the phagosome using the small molecule (R)-roscovitine restored the impaired phagosomal acidification of macrophages from CF patients [71]. TRPC6 is also known to regulate theCa^2+^-currents in Jurkat T-cells upon the T-cell receptor activation [73] or DAG stimulation [74]. Furthermore, mice with a systemic TRPC6 knockout produced lower levels of T-helper type 2 (Th2) cytokines (i.e., interleukin-5 (IL-5) and interleukin-13 (IL-13)) as compared with the control mice [75]. In addition, T-cell apoptosis was suppressed using the TRPC6 inhibitor SKF96365 in rats [76].

The nuclear translocation of the nuclear factor of activated T-cells (NFAT) is a crucial transcription factor for T-cell activation and B-cell development [77,78]. Interestingly, TRPC6-mediated Ca^2+^ influx in podocytes is known to lead to activation of Calcineurin as well as subsequently increased activity and the nuclear translocation of NFAT via a feed-forward loop [36]. Future studies should clarify if and how the TRPC6-mediated Ca^2+^ currents are involved in the immune cell functions via NFAT activation.

### 4.3. TRPC6 in the Endothelium

TRPC6 is also involved in inflammatory responses that involve the endothelium by promoting transendothelial-leukocyte migration. For example, TRPC6 activity has been shown to lead to endothelial cell contraction and, consequently, increased endothelial permeability [79,80]. Furthermore, endothelial TRPC6 is activated via an interaction with cluster of differentiation 31 (CD31) on leukocytes [81]. Activation of TRPC6 leads to trafficking of the lateral border recycling compartment (LBRC) to the leukocyte. LBRC subsequently supports the migration of leukocytes across the endothelial cell layer.

## 5. TRPC6-Mediated Calpain Activation in the Immune System

The cysteine protease Calpain plays a key role in the migration and chemotaxis of various types of immune cells, including neutrophils, eosinophils, dendritic cells and macrophages [82,83,84,85,86]. TRPC6-mediated Calpain activation might play an important role in the immunologic role of TRPC6, as TRPC6 has been shown to bind to and activate Calpain [87,88]. Calpain exerts its effect on immune cell migration via the degradation of the Talin protein family. Talin is a high-molecular-weight cytoskeletal protein, which links the actin cytoskeleton to integrins at the site of focal adhesions. Calpain-mediated Talin degradation has been shown to be the rate-limiting step during focal adhesion turnover, a key event during cell migration [89,90]. Significantly, it has been shown that TRPC6 also regulates podocyte cytoskeletal rearrangements and eventually leads to podocyte injury via the activation of Calpain [38]. This signalling pathway also appears to play a role in podocyte autophagy [91]. In addition to immune cell migration, Calpain also plays a key role in T-cell activation by activating the nuclear factor kappa-light-chain-enhancer of the activated B-cells (NF-kB) pathway [92,93]. Furthermore, Calpain might regulate T-cell activation by controlling Talin expression at the site of the immunological synapse between the antigen presenting cell and the T-cells [94,95,96,97]. Talin is known to orchestrate the actin dynamics at the immunological synapse, a crucial process for ensuring optimal T-cell activation [95,96]. Calpain-mediated Talin degradation at the immunological synapse might therefore interfere with T-cell activation. Finally, Calpain regulates interleukin-17 (IL-17) expression in T-lymphocytes and is involved in calcium-regulated NOX-independent NET formation and the phagocytic clearance of bacteria by macrophages [65,98,99,100,101]. This suggests that TRPC6 might exert a wide variety of interactions with the immune system via the activation of Calpain.

## 6. TRPC6-Mediated Calpain Activation in the Immune System

Increased TRPC6 expression and activity, either acquired or due to genetic mutations, have been shown to play key roles in the pathogenesis of glomerular diseases. Increased TRPC6 expression in podocytes and, consequently, an enhanced Ca^2+^ influx leads to cytoskeletal rearrangements, podocyte injury and eventually podocyte death.

### 6.1. TRPC6 and Immune Cell Infiltration into the Kidneys

An increasing number of studies also suggests that TRPC6 contributes to the development of glomerular injury by mediating immune cell infiltration in the kidneys. As discussed previously, TRPC6 plays an important role in regulating the adhesion of neutrophils by enhancing the activation of β2-integrins [45]. Furthermore, TRPC6 is crucial for neutrophil migration due to its role in regulating cytoskeletal remodelling [51,52]. Moreover, TRPC6 is involved in endothelial cell contraction, which might facilitate renal immune cell infiltration [79,80]. Based on these lines of evidence, TRPC6 might be involved in the pathogenesis of glomerular diseases that are characterized by glomerular infiltration of neutrophils, macrophages or T-cells, such as I/R, DN, and glomerulonephritis [102,103,104,105,106]. Indeed, neutrophil influx and renal damage upon I/R was reduced when bone marrow cells from TRPC6 knockout mice were transplanted in WT mice, as compared to WT mice transplanted with WT bone marrow cells [45]. Bone marrow cells from TRPC6 knockout mice showed decreased neutrophilic integrin activation, ICAM-1 binding, neutrophil adhesion and neutrophil influx into the kidneys upon I/R.

UUO is an experimental disease model that mimics renal fibrosis and is characterized by the infiltration of macrophages into the kidney [107,108]. The renal infiltration of immune cells is a direct consequence of the formation of tubulointerstitial fibrosis due to tubular pressure overload during UUO. The non-immunological role of TRPC6 in the pathogenesis of UUO has been shown previously as the inhibition of TRPC6 in interstitial fibroblasts using BTP2 decreased renal fibrosis [108]. Interestingly, several studies have also highlighted the important immunological role of TRPC6 in the pathogenesis of UUO. For example, a systemic TRPC6 knockout reduced the infiltration of macrophages and T-cells into the kidneys upon inducing UUO [43]. Furthermore, the TRPC6 inhibitor BI 749327 diminished the CD3+ T-cell infiltration in the kidneys after UUO [44]. In addition, Huangkui capsule, an herbal adjuvant therapy propagated for chronic kidney disease (CKD), reduced the influx of macrophages into the kidneys and consequently reduced the progression of renal fibrosis via the inhibition of TRPC6 [46].

Puromycin aminonucleoside (PAN)-induced nephrosis is a widely used experimental model that mimics glomerular injury. PAN directly damages the cytoskeleton of the podocytes, leading to glomerular injury and proteinuria [109,110]. In line with the central role of TRPC6 in cytoskeletal rearrangements during podocyte injury, podocyte-specific TRPC6 expression is indeed increased during PAN-nephrosis. Moreover, the TRPC6 knockout decreases proteinuria during PAN-nephrosis [42,111,112,113]. Notably, PAN-induced nephrosis is also associated with an increased glomerular influx of macrophages [42]. Also importantly, TRPC6 inactivation using CRISPR/Cas9 resulted in a reduced glomerular influx of macrophages as compared to WT littermates upon PAN-induced nephrosis [42]. Rats with an inactivated TRPC6 showed reduced glomerulosclerosis, podocyte foot process effacement and glomerular basement thickening.

Kidney inflammation is a crucial factor for the development of DKD, and DKD is characterized by the renal infiltration of the macrophages and T-cells [104]. Intriguingly, the immunosuppressive drug Tacrolimus inhibited the renal infiltration of the macrophages and subsequently inhibited glomerular injury in an experimental model of diabetic kidney disease. The underlying protective mechanism was at least partially attributed to the inhibition of the NFAT/TRPC6 pathway in proximal tubular cells, which eventually resulted in decreased macrophage infiltration [114].

**Figure 3:**
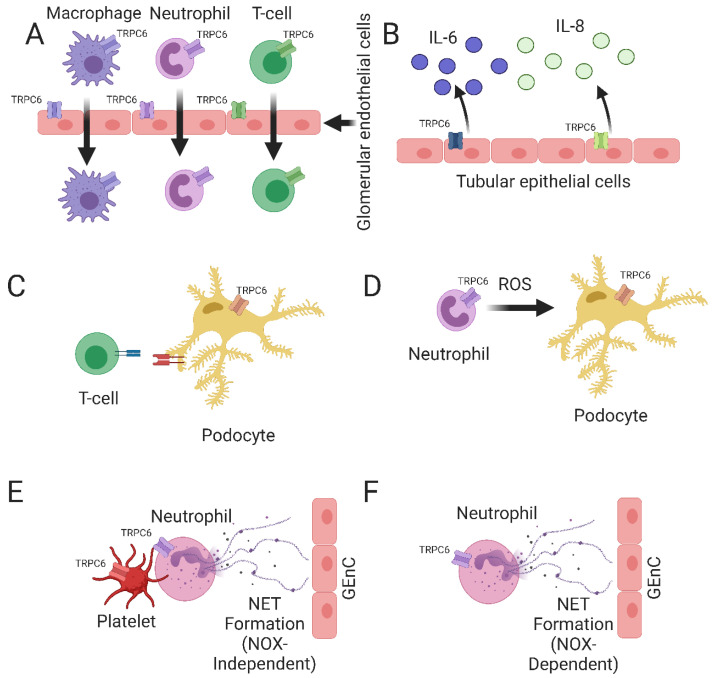
Proposed mechanisms regarding how TRPC6 is involved in immune-related kidney disorders. (**A**) Infiltration of macrophages, neutrophils and T-cells into the kidney, e.g., via increased integrin activation (via TRPC6 expressed by endothelial cells) and/or remodelling of the actin cytoskeleton (via TRPC6 expressed by immune cells) (**B**) Mediating tubulointerstitial inflammation by TRPC6 expressed by tubular epithelial cells via e.g., the production of pro-inflammatory cytokines IL-6 and IL-8 (**C**) T-cell activation via podocytic or glomerular endothelial cell antigen presentation. (**D**) ROS produced by neutrophils activate deleterious TRPC6 signalling in podocytes and subsequent podocyte cytoskeletal rearrangements and podocyte death (**E**) Increased neutrophil activation and subsequent NET formation via the NOX-independent pathway via aberrant platelet activation. (**F**) NOX activation and subsequent NOX-dependent NET formation by TRPC6 results in NET deposition on e.g., glomerular endothelial cells.

In summary, TRPC6 could play an important role in glomerular inflammation and tubulointerstitial fibrosis in several types of kidney diseases that are characterized by immune cell infiltration (Figure 3A and Table 2). Additional studies are still required to further elucidate the underlying mechanisms that would explain how TRPC6 is involved in immune cell infiltration. For example, by using cell-type-specific TRPC6 KO animal models, it can be better determined whether TRPC6 expressed by immune cells or TRPC6 expressed by e.g., endothelial cells is the main driver of immune cell infiltration in the kidneys.

### 6.2. TRPC6 and Tubulointerstitial Inflammation

In addition to immune cell invasion, TRPC6 also contributes to the progression of renal inflammation by promoting tubulointerstitial inflammation. Tubulointerstitial inflammation is a crucial event for the disease progression of several renal disorders, including DKD [138]. Enhanced glomerular TRPC6 expression is correlated with the increased secretion of the pro-inflammatory cytokines interleukin-6 (IL-6) and interleukin-8 (IL-8) by tubular epithelial cells [115,116]. Furthermore, a recent study showed that Tacrolimus could prevent tubulointerstitial inflammation and tubulointerstitial injury in experimental DKD via the inhibition of the NFAT-TRPC6 pathway [114]. In addition, TRPC6 expression in the kidneys of DKD patients correlated positively with tubulointerstitial inflammation [114]. Moreover, a systemic TRPC6 knockout during PAN-induced nephrosis resulted in reduced tubulointerstitial fibrosis, tubulointerstitial inflammation and tubular injury [41,42]. As PAN-induced nephrosis is a glomerular injury model, reduced tubulointerstitial fibrosis could be a secondary result of reduced glomerular injury, decreased proteinuria and tubular protein overload after TRPC6 KO. Finally, a systemic TRPC6 knock-out or TRPC6 inhibition via SAR7334 prevented apoptosis of the proximal tubular cells during I/R and eventually prevented further progression of renal inflammation [25]. The underlying mechanism unveiled that TRPC6 normally inhibits cytoprotective autophagy in proximal tubular cells upon I/R-related oxidative stress. The absence of TRPC6 resulted in enhanced cytoprotective autophagy and thereby improved the survival of tubular cells. Some controversy remains about the role of TRPC6 in the context of tubulointerstitial inflammation; two other studies showed that TRPC6 protects against I/R progression via the inhibition of the necroptosis of the tubular epithelial cells [117,118]. A possible explanation for these opposing results might be that different experimental models were used to mimic I/R. TRPC6 also plays an important role in immune-mediated kidney injuries by regulating tubulointerstitial inflammation and the further development of kidney inflammation (Figure 3B and Table 2). Whether this effect is mediated by the TRPC6 expressed by glomerular cells and tubuli or by the TRPC6 expressed by immune cells can be investigated in future studies using methods such as chimeric bone marrow transplantation models.

### 6.3. TRPC6 and Antigen Presentation by Podocytes

In addition to their known role in glomerular filtration, podocytes might also contribute to the development of glomerular inflammation. It was elegantly proven by Goldwich et al. that podocytes are professional antigen-presenting cells by the expression of MHC-II and activation of the CD4 and CD8+ cells [119]. The contribution of the podocytic antigen presentation to the development of renal inflammation was recently demonstrated by a podocyte-specific knockout model of the neonatal Fc receptor (FcRn). Notably, FcRn can contribute to antigen presentation by the most efficient professional antigen-presenting cells, i.e., dendritic cells [120]. Podocyte-specific FcRn knockout decreased interleukin-6 (IL-6) production in podocytes and reduced disease progression in a nephrotoxic serum-induced nephritis disease model [121,122]. TRPC6 might be involved in the process of antigen presentation in podocytes via STIM1. STIM1-mediated Ca^2+^ entry leads to increased TRPC6 externalization and Ca^2+^ entry via TRPC6 [139], while STIM1-mediated Ca^2+^ entry has been shown to be crucial for antigen cross-presentation in dendritic cells [123,124]. Increased expression of podocytic TRPC6, as observed during I/R and DN, might lead to enhanced antigen presentation by podocytes, increased activation of the immune system and the progression of renal inflammation [33,108,112,140,141] (Figure 3C and Table 2). Future studies should elucidate if and how TRPC6 contributes to podocyte antigen presentation and, subsequently, glomerular inflammation. For example, podocyte- specific TRPC6 KO animal models should be developed to investigate the contribution of TRPC6 to antigen presentation in podocytes during renal inflammation.

### 6.4. Activation of Deleterious TRPC6 Signalling by Neutrophil-Derived ROS

Immune-cell-specific TRPC6 might also cause further kidney injury by activating the deleterious TRPC6 signalling pathway in the podocyte. As discussed previously, the NOX complex activation and the subsequent ROS production by neutrophils is dependent on TRPC6 channel activity [125]. Interestingly, several studies have shown that ROS increases the surface expression and activity of TRPC6 in podocytes [31,49,50,126]. These findings might suggest that elevated extracellular ROS production, for example by neutrophils, might directly lead to enhanced TRPC6 expression and activity in podocytes (Figure 3D and Table 2). The subsequently increased deleterious TRPC6 signalling activity in podocytes aggravates the already damaging effect of the glomerular inflammation. For example, during CKD, which is characterized by an increased neutrophil influx in the kidneys and chronic kidney inflammation [142,143], ROS-induced TRPC6 activation in podocytes might play an important pathogenic role. ROS-induced TRPC6 activation might also play an important role in the pathophysiology of DKD. For example, it is known that hyperglycemia, as observed during DKD, results in the elevated activation of Angiotensin II (Ang II) [144]. Increased activation of Ang II might lead to the enhanced synthesis of ROS via NAPDH Oxidase 4 (NOX4) activation. Elevated ROS synthesis results in an increased TRPC6 activation and the further disease progression of DKD [49,145,146,147]. In this way, TRPC6 contributes to the disease progression of DKD in addition to its abovementioned role in immune cell infiltration.

### 6.5. Pro-Inflammatory Role of TRPC6 in the Context of Lupus Nephritis

TRPC6 might also play a pro-inflammatory role in glomerular diseases that are characterized by the deposition of immune complexes on the GBM, e.g., systemic lupus erythematosus (SLE) [148]. These immune complexes trigger inflammation and eventually lead to tissue damage. A frequent and dangerous complication of SLE is the development of lupus nephritis (LN) in up to 50–60% of the patients, which in many cases leads to renal failure within a few years [149]. Intriguingly, a single nucleotide polymporphism (SNP) in TRPC6 altered the functionality of the peripheral blood mononuclear cells (PBMC) of SLE patients [150]. PBMC from SLE patients with this SNP in TRPC6 were more dependent on TRPC6 for Ca^2+^ currents. Furthermore, interleukin-17 (IL-17) synthesis in PBMC from SLE patients, but not from healthy subjects, relied on the TRPC6-mediated Ca^2+^ gradients. Although the SNP in TRPC6 was primarily associated with neurological complications, these findings highlight the involvement of TRPC6 in the pathogenesis of SLE and potentially in LN. Future studies should determine if other SNPs in TRPC6 are also linked to LN.

Urine-derived podocytes from SLE patients were also characterized by elevated TRPC6 mRNA levels [151]. The increased TRPC6 mRNA levels correlated with both the severity of the LN and the invasion of the CD8+ T-cells, macrophages and B-cells in the kidneys. However, it remains uncertain whether the elevated TRPC6 expression in podocytes is mechanistically involved in the immune pathogenesis of LN, as it can also be elevated due to the activation of a final common pathway in the podocyte injury. It is important to note that neutrophils from SLE patients are characterized by enhanced ROS production [152]. As discussed previously, enhanced neutrophil-derived ROS production might lead to an increased podocyte-specific TRPC6 expression, deleterious TRPC6 signalling and, eventually, podocyte death.

TRPC6 might also be functionally involved in the pathogenesis of LN at the levels of platelets. CF patients are characterized by hyperactive platelets, which lead to excessive neutrophil activation, NOX-independent NET formation and lung inflammation [153,154]. The TRPC6 knockout prevents platelet hyperactivation, NET formation and the progression of lung inflammation [69,129]. Platelets are known to be potent inducers of NET formation via the NOX-independent pathway, thereby suggesting that TRPC6 stimulates NET formation via the NOX-independent pathway [155,156]. However, as TRPC6 is also linked to NOX-dependent NET formation the effect of TRPC6 KO on reduced NET formation could be caused via both the NOX-dependent and the NOX-independent pathways [48].

Notably, platelets also play a key role during the pathogenesis of LN, and SLE patients are also characterized by hyperactive platelets and high levels of NOX-independent NET formation [130,131]. Furthermore, glomerular NET deposition correlates with the disease progression of LN [132], while the TRPC6 activity is fundamental for platelet activation [133,134,135,136,137]. Future studies should elucidate whether TRPC6 is responsible for platelet hyperactivation in the context of SLE, increased neutrophil activation, increased NET formation and the disease progression of LN using cell-type specific TRPC6 knockout animal models.

Most studies suggest that the role of TRPC6 in the context of immune-mediated kidney diseases is a pathogenic one. However, one study showed that acute activation of TRPC6 in the podocyte protected against the development of acute complement-mediated glomerular disease [157]. Mice overexpressing TRPC6 in podocytes demonstrated decreased podocyte foot effacement and proteinuria as compared to systemic TRPC6 knockout mice in nephrotoxic serum-induced nephritis. These results might suggest that short-term TRPC6 activation in podocytes is important for regulating a controlled immune response and preventing further organ damage. By contrast, chronic TRPC6 activation leads to an excessive inflammatory response, the activation of deleterious TRPC6 signalling and the progression of podocyte injury.

A similar controversial role of TRPC6-mediated Ca^2+^ entry in the context of immune-related glomerular diseases has been described for type 1 diabetes. In one experimental model of type 1 diabetes performed on Akita mice, TRPC6 KO resulted in increased insulin resistance and subsequently caused increased glomerular injury and disease progression [158]. By contrast, in a different experimental model for type 1 diabetes performed on, Dahl-sensitive rats, the KO of the NOX4 resulted in a reduced Ca^2+^-influx and decreased disease progression [147]. A potential explanation regarding the context-specific role of TRPC6 per disease model was given by a study showing that the NOX expression was increased in mesangial cells and podocytes in DKD [159,160,161]. In sharp contrast, the NOX expression was decreased in the proximal tubular cells in experimental models of chronic kidney disease [162].

## 7. Concluding Remarks

In conclusion, TRPC6 is known to be an important regulator of immune cell function. In addition, podocyte TRPC6 is well-known as being involved in podocyte injuries and glomerular disease by mediating deleterious intracellular signaling pathways and podocyte cytoskeletal rearrangements. In this review, we have highlighted how TRPC6 can also function as a key pathogenic mediator in inflammatory kidney diseases (Figure 3) via at least the following six potential mechanisms: (1) regulation of the immune cell infiltration of the kidneys, (2) mediation of tubulointerstitial inflammation, (3) activation of the immune cells secondary to antigen presentation by podocytes, (4) induction of ROS-activated deleterious TRPC6 signaling in podocytes, (5) stimulation of platelet hyperactivation and subsequent NET formation via the NOX-independent pathway and (6) NET formation via the NOX-dependent pathway (Figure 3 and Table 2). A better understanding of the role of TRPC6 in inflammatory kidney diseases might open new therapeutic avenues for the treatment of immune-mediated kidney injury by the pharmacological modulation of TRPC6 activity. Notably, the first clinical trial using a TRPC6 inhibitor (i.e., BI 764198) for the treatment of FSGS is currently ongoing [163]. It would be highly interesting to learn from this study if the (potential) therapeutic effect of BI 764198 on the development of FSGS is mediated primarily via a direct effect on the podocyte or via the therapeutic inhibition of the immune system.

## Figures and Tables

**Table 1:** Overview of the roles of TRPC6 in different immune cell types.

Expressed in	Function	References
Neutrophils	SOCE Superoxide production Chemotaxis/migration Adhesion NOX-dependent NET formation NOX-independent NET formation	[48] [48] [51,52,53] [45] [48] [69,70]
Macrophages	Phagocytosis	[71]
Mast cell	Degranulation	[72]
T-cells	ROCE Cytokine production Apoptosis	[73,74] [75] [76]

ROCE; receptor-operated Calcium entry, SOCE; store-operated Calcium entry.

**Table 2:** Overview of the (putative) roles of TRPC6 in immune-related kidney disorders DKD and UUO.

(Putative) Mechanism	(Putatively) Involved in the Pathogenesis of	References
Immune cell infiltration	I/R, UUO, PAN-induced nephrosis, DN	[42,43,44,45,46]
Tubulointerstitial inflammation	DN, PAN-induced nephrosis, I/R	[25,41,42,114,115,116,117,118]
Antigen presentation by podocytes	I/R, DN	[16,119,120,121,122,123,124]
ROS-induced overexpression of TRPC6	CKD	[31,49,50,125,126,127,128]
Platelet hyperactivation and subsequent NOX-independent NET formation	LN	[69,70,129,130,131,132,133,134,135,136,137]

CKD; chronic kidney disease DN; diabetic kidney disease, I/R; Ischemia/reperfusion injury, LN; lupus nephritis, PAN; Puromycin aminonucleoside, UUO; unilateral ureteral obstruction.

## Data Availability

Not applicable.

## References

[1] Montell C., Birnbaumer L., Flockerzi V., Bindels R.J., Bruford E.A., Caterina M.J., Clapham D.E., Harteneck C., Heller S., Julius D. (2002). A Unified Nomenclature for the Superfamily of TRP Cation Channels. Mol. Cell.

[2] Vazquez G., Wedel B.J., Aziz O., Trebak M., Putney J.W. (2004). The mammalian TRPC cation channels. Biochim. Biophys Acta (BBA)-Mol. Cell Res..

[3] Dietrich A., Kalwa H., Rost B.R., Gudermann T. (2005). The diacylgylcerol-sensitive TRPC3/6/7 subfamily of cation channels: Functional characterization and physiological relevance. Pflugers Arch..

[4] Vannier B., Peyton M., Boulay G., Brown D., Qin N., Jiang M., Zhu X., Birnbaumer L. (1999). Mouse trp2, the homologue of the human trpc2 pseudogene, encodes mTrp2, a store depletion-activated capacitative Ca^2+^ entry channel. Proc. Natl. Acad. Sci. USA.

[5] Hofmann T., Schaefer M., Schultz G., Gudermann T. (2002). Subunit composition of mammalian transient receptor potential channels in living cells. Proc. Natl. Acad. Sci. USA.

[6] Goel M., Sinkins W.G., Schilling W.P. (2002). Selective Association of TRPC Channel Subunits in Rat Brain Synaptosomes. J. Biol. Chem..

[7] Villereal M.L. (2006). Mechanism and functional significance of TRPC channel multimerization. Semin. Cell Dev. Biol..

[8] Cayouette S., Lussier M.P., Mathieu E.-L., Bousquet S.M., Boulay G. (2004). Exocytotic Insertion of TRPC6 Channel into the Plasma Membrane upon Gq Protein-coupled Receptor Activation. J. Biol. Chem..

[9] Chaudhuri P., Rosenbaum M.A., Sinharoy P., Damron D.S., Birnbaumer L., Graham L.M. (2016). Membrane translocation of TRPC6 channels and endothelial migration are regulated by calmodulin and PI3 kinase activation. Proc. Natl. Acad. Sci. USA.

[10] Dietrich A., Mederos y Schnitzler M., Emmel J., Kalwa H., Hofmann T., Gudermann T. (2003). N-Linked Protein Glycosylation Is a Major Determinant for Basal TRPC3 and TRPC6 Channel Activity. J. Biol. Chem..

[11] Hofmann T., Obukhov A.G., Schaefer M., Harteneck C., Gudermann T., Schultz G. (1999). Direct activation of human TRPC6 and TRPC3 channels by diacylglycerol. Nature.

[12] Okada T., Inoue R., Yamazaki K., Maeda A., Kurosaki T., Yamakuni T., Tanaka I., Shimizu S., Ikenaka K., Imoto K. (1999). Molecular and Functional Characterization of a Novel Mouse Transient Receptor Potential Protein Homologue TRP7. J. Biol. Chem..

[13] Balla T. (2010). Putting G protein–coupled receptor-mediated activation of phospholipase C in the limelight. J. Gen. Physiol..

[14] Venkatachalam K., Zheng F., Gill D.L. (2003). Regulation of Canonical Transient Receptor Potential (TRPC) Channel Function by Diacylglycerol and Protein Kinase C. J. Biol. Chem..

[15] Cheng K.T., Ong H.L., Liu X., Ambudkar I.S. (2013). Contribution and regulation of TRPC channels in store-operated Ca^2+^ Entry. Current Topics in Membranes.

[16] Chen W., Thielmann I., Gupta S., Subramanian H., Stegner D., van Kruchten R., Dietrich A., Gambaryan S., Heemskerk J.W.M., Hermanns H.M. (2014). Orai1-induced store-operated Ca^2+^ entry enhances phospholipase activity and modulates canonical transient receptor potential channel 6 function in murine platelets. J. Thromb. Haemost..

[17] Dietrich A., Gudermann T. (2014). TRPC6: Physiological function and pathophysiological relevance. Handb. Exp. Pharmacol..

[18] Dietrich A., Schnitzler M.M.Y., Gollasch M., Gross V., Storch U., Dubrovska G., Obst M., Yildirim E., Salanova B., Kalwa H. (2005). Increased Vascular Smooth Muscle Contractility in TRPC6−/− Mice. Mol. Cell. Biol..

[19] Malczyk M., Erb A., Veith C., Ghofrani H.A., Schermuly R.T., Gudermann T., Dietrich A., Weissmann N., Sydykov A. (2017). The Role of Transient Receptor Potential Channel 6 Channels in the Pulmonary Vasculature. Front. Immunol..

[20] Tsvilovskyy V.V., Zholos A.V., Aberle T., Philipp S.E., Dietrich A., Zhu M.X., Birnbaumer L., Freichel M., Flockerzi V. (2009). Deletion of TRPC4 and TRPC6 in Mice Impairs Smooth Muscle Contraction and Intestinal Motility In Vivo. Gastroenterology.

[21] Koitabashi N., Aiba T., Hesketh G.G., Rowell J., Zhang M., Takimoto E., Tomaselli G.F., Kass D.A. (2010). Cyclic GMP/PKG-dependent inhibition of TRPC6 channel activity and expression negatively regulates cardiomyocyte NFAT activation: Novel mechanism of cardiac stress modulation by PDE5 inhibition. J. Mol. Cell. Cardiol..

[22] Kuwahara K., Wang Y., McAnally J., Richardson J.A., Bassel-Duby R., Hill J.A., Olson E.N. (2006). TRPC6 fulfills a calcineurin signaling circuit during pathologic cardiac remodeling. J. Clin. Investig..

[23] Goel M., Sinkins W.G., Zuo C.-D., Estacion M., Schilling W.P. (2006). Identification and localization of TRPC channels in the rat kidney. Am. J. Physiol. Physiol..

[24] Sours S., Du J., Chu S., Ding M., Zhou X.J., Ma R. (2006). Expression of canonical transient receptor potential (TRPC) proteins in human glomerular mesangial cells. Am. J. Physiol. Physiol..

[25] Hou X., Xiao H., Zhang Y., Zeng X., Huang M., Chen X., Birnbaumer L., Liao Y. (2018). Transient receptor potential channel 6 knockdown prevents apoptosis of renal tubular epithelial cells upon oxidative stress via autophagy activation. Cell Death Dis..

[26] Reiser J., Polu K.R., Möller C.C., Kenlan P., Altintas M.M., Wei C., Faul C., Herbert S., Villegas I., Avila-Casado C. (2005). TRPC6 is a glomerular slit diaphragm-associated channel required for normal renal function. Nat. Genet..

[27] Schlöndorff J.S., Pollak M.R. (2006). TRPC6 in glomerular health and disease: What we know and what we believe. Semin. Cell Dev. Biol..

[28] Huber T.B., Köttgen M., Schilling B., Walz G., Benzing T. (2001). Interaction with Podocin Facilitates Nephrin Signaling. J. Biol. Chem..

[29] Jiang L., Ding J., Tsai H., Li L., Feng Q., Miao J., Fan Q. (2011). Over-expressing transient receptor potential cation channel 6 in podocytes induces cytoskeleton rearrangement through increases of intracellular Ca^2+^ and RhoA activation. Exp. Biol. Med..

[30] Kanda S., Harita Y., Shibagaki Y., Sekine T., Igarashi T., Inoue T., Hattori S., Chen R.-H., Brady D.M., Smith D. (2011). Tyrosine phosphorylation–dependent activation of TRPC6 regulated by PLC-γ1 and nephrin: Effect of mutations associated with focal segmental glomerulosclerosis. Mol. Biol. Cell.

[31] Kim E.Y., Anderson M., Wilson C., Hagmann H., Benzing T., Dryer S.E. (2013). NOX2 interacts with podocyte TRPC6 channels and contributes to their activation by diacylglycerol: Essential role of podocin in formation of this complex. Am. J. Physiol. Physiol..

[32] Tomilin V., Mamenko M., Zaika O., Pochynyuk O. (2015). Role of renal TRP channels in physiology and pathology. Semin. Immunopathol..

[33] Winn M.P., Conlon P.J., Lynn K.L., Farrington M.K., Creazzo T., Hawkins A.F., Daskalakis N., Kwan S.Y., Ebersviller S., Burchette J.L. (2005). A Mutation in the TRPC6 Cation Channel Causes Familial Focal Segmental Glomerulosclerosis. Science.

[34] Daskalakis N., Winn M.P. (2006). Focal and segmental glomerulosclerosis. Cell. Mol. Life Sci..

[35] Endlich N., Endlich K. (2012). The Challenge and Response of Podocytes to Glomerular Hypertension. Semin. Nephrol..

[36] Nijenhuis T., Sloan A.J., Hoenderop J.G., Flesche J., van Goor H., Kistler A.D., Bakker M., Bindels R.J., de Boer R.A., Möller C.C. (2011). Angiotensin II Contributes to Podocyte Injury by Increasing TRPC6 Expression via an NFAT-Mediated Positive Feedback Signaling Pathway. Am. J. Pathol..

[37] Wang Q., Tian X., Wang Y., Wang Y., Li J., Zhao T., Li P. (2020). Role of Transient Receptor Potential Canonical Channel 6 (TRPC6) in Diabetic Kidney Disease by Regulating Podocyte Actin Cytoskeleton Rearrangement. J. Diabetes Res..

[38] Verheijden K.A., Sonneveld R., Bebber M.B.-V., Wetzels J.F., van der Vlag J., Nijenhuis T. (2018). The Calcium-Dependent Protease Calpain-1 Links TRPC6 Activity to Podocyte Injury. J. Am. Soc. Nephrol..

[39] Sonneveld R., Ferrè S., Hoenderop J.G., Dijkman H.B., Berden J.H., Bindels R.J., Wetzels J.F., van der Vlag J., Nijenhuis T. (2013). Vitamin D Down-Regulates TRPC6 Expression in Podocyte Injury and Proteinuric Glomerular Disease. Am. J. Pathol..

[40] Staruschenko A., Spires D., Palygin O. (2019). Role of TRPC6 in Progression of Diabetic Kidney Disease. Curr. Hypertens. Rep..

[41] Wang L., Jirka G., Rosenberg P.B., Buckley A.F., Gomez J.A., Fields T.A., Winn M.P., Spurney R.F. (2015). Gq signaling causes glomerular injury by activating TRPC6. J. Clin. Investig..

[42] Kim E.Y., Shotorbani P.Y., Dryer S.E. (2018). Trpc6 inactivation confers protection in a model of severe nephrosis in rats. J. Mol. Med..

[43] Kong W., Haschler T.N., Nürnberg B., Krämer S., Gollasch M., Markó L. (2019). Renal Fibrosis, Immune Cell Infiltration and Changes of TRPC Channel Expression after Unilateral Ureteral Obstruction in Trpc6−/− Mice. Cell. Physiol. Biochem..

[44] Lin B.L., Matera D., Doerner J.F., Zheng N., del Camino D., Mishra S., Bian H., Zeveleva S., Zhen X., Blair N.T. (2019). In vivo selective inhibition of TRPC6 by antagonist BI 749327 ameliorates fibrosis and dysfunction in cardiac and renal disease. Proc. Natl. Acad. Sci. USA.

[45] Lindemann O., Rossaint J., Najder K., Schimmelpfennig S., Hofschröer V., Wälte M., Fels B., Oberleithner H., Zarbock A., Schwab A. (2020). Intravascular adhesion and recruitment of neutrophils in response to CXCL1 depends on their TRPC6 channels. J. Mol. Med..

[46] Gu L.-F., Ge H.-T., Zhao L., Wang Y.-J., Zhang F., Tang H.-T., Cao Z.-Y., Yu B.-Y., Chai C.-Z. (2020). Huangkui Capsule Ameliorates Renal Fibrosis in a Unilateral Ureteral Obstruction Mouse Model Through TRPC6 Dependent Signaling Pathways. Front. Pharmacol..

[47] Birben E., Sahiner U.M., Sackesen C., Erzurum S., Kalayci O. (2012). Oxidative Stress and Antioxidant Defense. World Allergy Organ. J..

[48] Bréchard S., Melchior C., Plançon S., Schenten V., Tschirhart E. (2008). Store-operated Ca^2+^ channels formed by TRPC1, TRPC6 and Orai1 and non-store-operated channels formed by TRPC3 are involved in the regulation of NADPH oxidase in HL-60 granulocytes. Cell Calcium.

[49] Anderson M., Roshanravan H., Khine J., Dryer S.E. (2013). Angiotensin II Activation of TRPC6 Channels in Rat Podocytes Requires Generation of Reactive Oxygen Species. J. Cell. Physiol..

[50] Kim E.Y., Khayyat N.H., Dryer S.E. (2018). Mechanisms underlying modulation of podocyte TRPC6 channels by suPAR: Role of NADPH oxidases and Src family tyrosine kinases. Biochim. Et Biophys. Acta (BBA)-Mol. Basis Dis..

[51] Lindemann O., Umlauf D., Frank S., Schimmelpfennig S., Bertrand J., Pap T., Hanley P.J., Fabian A., Dietrich A., Schwab A. (2013). TRPC6 Regulates CXCR2-Mediated Chemotaxis of Murine Neutrophils. J. Immunol..

[52] Damann N., Owsianik G., Li S., Poll C., Nilius B. (2008). The calcium-conducting ion channel transient receptor potential canonical 6 is involved in macrophage inflammatory protein-2-induced migration of mouse neutrophils. Acta Physiol..

[53] Lindemann O., Strodthoff C., Horstmann M., Nielsen N., Jung F., Schimmelpfennig S., Heitzmann M., Schwab A. (2015). TRPC1 regulates fMLP-stimulated migration and chemotaxis of neutrophil granulocytes. Biochim. et Biophys. Acta (BBA)-Mol. Cell Res..

[54] Díaz-Godínez C., Carrero J.C. (2019). The state of art of neutrophil extracellular traps in protozoan and helminthic infections. Biosci. Rep..

[55] Boeltz S., Amini P., Anders H.-J., Andrade F., Bilyy R., Chatfield S., Cichon I., Clancy D.M., Desai J., Dumych T. (2019). To NET or not to NET:current opinions and state of the science regarding the formation of neutrophil extracellular traps. Cell Death Differ..

[56] Kaplan M.J., Radic M. (2012). Neutrophil Extracellular Traps: Double-Edged Swords of Innate Immunity. J. Immunol..

[57] Yang H., Biermann M.H., Brauner J.M., Liu Y., Zhao Y., Herrmann M. (2016). New Insights into Neutrophil Extracellular Traps: Mechanisms of Formation and Role in Inflammation. Front. Immunol..

[58] Pieterse E., Rother N., Yanginlar C., Gerretsen J., Boeltz S., Munoz L.E., Herrmann M., Pickkers P., Hilbrands L.B., van der Vlag J. (2018). Cleaved N-terminal histone tails distinguish between NADPH oxidase (NOX)-dependent and NOX-independent pathways of neutrophil extracellular trap formation. Rheumatology.

[59] Yipp B.G., Kubes P. (2013). NETosis: How vital is it?. Blood.

[60] Cortjens B., de Boer O.J., De Jong R., Antonis A.F., Sabogal Piñeros Y.S., Lutter R., Van Woensel J.B., Bem R.A. (2016). Neutrophil extracellular traps cause airway obstruction during respiratory syncytial virus disease. J. Pathol..

[61] Garishah F.M., Rother N., Riswari S.F., Alisjahbana B., Overheul G.J., van Rij R.P., van der Ven A., van der Vlag J., de Mast Q. (2021). Neutrophil Extracellular Traps in Dengue Are Mainly Generated NOX-Independently. Front. Immunol..

[62] Strandin T., Mäkelä S., Mustonen J., Vaheri A. (2018). Neutrophil Activation in Acute Hemorrhagic Fever with Renal Syndrome Is Mediated by Hantavirus-Infected Microvascular Endothelial Cells. Front. Immunol..

[63] de Bont C.M., Koopman W.J., Boelens W.C., Pruijn G.J. (2018). Stimulus-dependent chromatin dynamics, citrullination, calcium signalling and ROS production during NET formation. Biochim. et Biophys. Acta (BBA)-Mol. Cell Res..

[64] De Samber B., Niemiec M.J., Laforce B., Garrevoet J., Vergucht E., De Rycke R., Cloetens P., Urban C.F., Vincze L. (2016). Probing Intracellular Element Concentration Changes during Neutrophil Extracellular Trap Formation Using Synchrotron Radiation Based X-Ray Fluorescence. PLoS ONE.

[65] Francis R.J., E Butler R., Stewart G.R. (2014). Mycobacterium tuberculosis ESAT-6 is a leukocidin causing Ca2+ influx, necrosis and neutrophil extracellular trap formation. Cell Death Dis..

[66] Kenny E.F., Herzig A., Krüger R., Muth A., Mondal S., Thompson P.R., Brinkmann V., von Bernuth H., Zychlinsky A. (2017). Diverse stimuli engage different neutrophil extracellular trap pathways. eLife.

[67] Gupta A.K., Giaglis S., Hasler P., Hahn S. (2014). Efficient Neutrophil Extracellular Trap Induction Requires Mobilization of Both Intracellular and Extracellular Calcium Pools and Is Modulated by Cyclosporine A. PLoS ONE.

[68] Douda D.N., Khan M.A., Grasemann H., Palaniyar N. (2015). SK3 channel and mitochondrial ROS mediate NADPH oxidase-independent NETosis induced by calcium influx. Proc. Natl. Acad. Sci. USA.

[69] Robledo-Avila F.H., de Dios Ruiz-Rosado J., Brockman K.L., Kopp B.T., Amer A.O., McCoy K., Bakaletz L.O., Partida-Sanchez S. (2018). Dysregulated Calcium Homeostasis in Cystic Fibrosis Neutrophils Leads to Deficient Antimicrobial Responses. J. Immunol..

[70] Xu S., Zeng F., Boulay G., Grimm C., Harteneck C., Beech D.J. (2005). Block of TRPC5 channels by 2-aminoethoxydiphenyl borate: A differential, extracellular and voltage-dependent effect. Br. J. Pharmacol..

[71] Riazanski V., Gabdoulkhakova A.G., Boynton L.S., Eguchi R.R., Deriy L.V., Hogarth D.K., Loaëc N., Oumata N., Galons H., Brown M.E. (2015). TRPC6 channel translocation into phagosomal membrane augments phagosomal function. Proc. Natl. Acad. Sci. USA.

[72] Sanchez-Miranda E., Ibarra-Sanchez A., Gonzalez-Espinosa C. (2010). Fyn kinase controls FcεRI receptor-operated calcium entry necessary for full degranulation in mast cells. Biochem. Biophys. Res. Commun..

[73] Tseng P.-H., Lin H.-P., Hu H., Wang C., Zhu M.X., Chen C.-S. (2004). The Canonical Transient Receptor Potential 6 Channel as a Putative Phosphatidylinositol 3,4,5-Trisphosphate-Sensitive Calcium Entry System. Biochemistry.

[74] Carrillo C., Hichami A., Andreoletti P., Cherkaoui-Malki M., Cavia M.d.M., Abdoul-Azize S., Alonso-Torre S.R., Khan N.A. (2012). Diacylglycerol-containing oleic acid induces increases in [Ca2+]i via TRPC3/6 channels in human T-cells. Biochim. Et Biophys. Acta (BBA)-Mol. Cell Biol. Lipids.

[75] Sel S., Rost B.R., Yildirim A.Ö., Sel B., Kalwa H., Fehrenbach H., Renz H., Gudermann T., Dietrich A. (2008). Loss of classical transient receptor potential 6 channel reduces allergic airway response. Clin. Exp. Allergy.

[76] Wu Q.-Y., Sun M.-R., Wu C.-L., Li Y., Du J.-J., Zeng J.-Y., Bi H.-L., Sun Y.-H. (2015). Activation of calcium-sensing receptor increases TRPC3/6 expression in T lymphocyte in sepsis. Mol. Immunol..

[77] Hogan P.G. (2017). Calcium–NFAT transcriptional signalling in T cell activation and T cell exhaustion. Cell Calcium.

[78] Giampaolo S., Wójcik G., Klein-Hessling S., Serfling E., Patra A.K. (2018). B cell development is critically dependent on NFATc1 activity. Cell. Mol. Immunol..

[79] Kini V., Chavez A., Mehta D. (2010). A New Role for PTEN in Regulating Transient Receptor Potential Canonical Channel 6-mediated Ca^2+^ Entry, Endothelial Permeability, and Angiogenesis *. J. Biol. Chem..

[80] Singh I., Knezevic N., Ahmmed G.U., Kini V., Malik A.B., Mehta D. (2007). Gαq-TRPC6-mediated Ca^2+^ Entry Induces RhoA Activation and Resultant Endothelial Cell Shape Change in Response to Thrombin. J. Biol. Chem..

[81] Weber E.W., Han F., Tauseef M., Birnbaumer L., Mehta D., Muller W.A. (2015). TRPC6 is the endothelial calcium channel that regulates leukocyte transendothelial migration during the inflammatory response. J. Exp. Med..

[82] Calle Y., Carragher N.O., Thrasher A.J., Jones G.E. (2006). Inhibition of calpain stabilises podosomes and impairs dendritic cell motility. J. Cell Sci..

[83] Lokuta M.A., Nuzzi P.A., Huttenlocher A. (2003). Calpain regulates neutrophil chemotaxis. Proc. Natl. Acad. Sci. USA.

[84] Nuzzi P.A., Senetar M.A., Huttenlocher A. (2007). Asymmetric Localization of Calpain 2 during Neutrophil Chemotaxis. Mol. Biol. Cell.

[85] Zuo J., Hu Z., Liu T., Chen C., Tao Z., Chen S., Li F. (2018). Calpeptin attenuates cigarette smoke-induced pulmonary inflammation via suppressing calpain/IκBα signaling in mice and BEAS-2B cells. Pathol.-Res. Pract..

[86] Roberts R.E., Hallett M.B. (2019). Neutrophil Cell Shape Change: Mechanism and Signalling during Cell Spreading and Phagocytosis. Int. J. Mol. Sci..

[87] Ji J., Su L., Liu Z. (2016). Critical role of calpain in inflammation. Biomed. Rep..

[88] Farmer L.K., Rollason R., Whitcomb D.J., Ni L., Goodliff A., Lay A.C., Birnbaumer L., Heesom K.J., Xu S.-Z., Saleem M.A. (2019). TRPC6 Binds to and Activates Calpain, Independent of Its Channel Activity, and Regulates Podocyte Cytoskeleton, Cell Adhesion, and Motility. J. Am. Soc. Nephrol..

[89] Franco S.J., Rodgers M.A., Perrin B.J., Han J., Bennin D.A., Critchley D.R., Huttenlocher A. (2004). Calpain-mediated proteolysis of talin regulates adhesion dynamics. Nature.

[90] Chinthalapudi K., Rangarajan E.S., Izard T. (2018). The interaction of talin with the cell membrane is essential for integrin activation and focal adhesion formation. Proc. Natl. Acad. Sci. USA.

[91] Salemkour Y., Yildiz D., Dionet L., Hart D.C., Verheijden K.A., Saito R., Mahtal N., Delbet J.-D., Letavernier E., Rabant M. (2023). Podocyte Injury in Diabetic Kidney Disease in Mouse Models Involves TRPC6-mediated Calpain Activation Impairing Autophagy. J. Am. Soc. Nephrol..

[92] Mikosik A., Jasiulewicz A., Daca A., Henc I., Frąckowiak J.E., Ruckemann-Dziurdzińska K., Foerster J., Le Page A., Bryl E., Fulop T. (2016). Roles of calpain-calpastatin system (CCS) in human T cell activation. Oncotarget.

[93] Schaecher K., Goust J.-M., Banik N.L. (2004). The Effects of Calpain Inhibition on IkBα Degradation After Activation of PBMCs: Identification of the Calpain Cleavage Sites. Neurochem. Res..

[94] Chen L., Flies D.B. (2013). Molecular mechanisms of T cell co-stimulation and co-inhibition. Nat. Rev. Immunol..

[95] Jankowska K.I., Williamson E.K., Roy N.H., Blumenthal D., Chandra V., Baumgart T., Burkhardt J.K. (2018). Integrins Modulate T Cell Receptor Signaling by Constraining Actin Flow at the Immunological Synapse. Front. Immunol..

[96] Comrie W.A., Babich A., Burkhardt J.K. (2015). F-actin flow drives affinity maturation and spatial organization of LFA-1 at the immunological synapse. J. Cell Biol..

[97] Klann J.E., Remedios K.A., Kim S.H., Metz P.J., Lopez J., Mack L.A., Zheng Y., Ginsberg M.H., Petrich B.G., Chang J.T. (2017). Talin Plays a Critical Role in the Maintenance of the Regulatory T Cell Pool. J. Immunol..

[98] Kumar V., Everingham S., Hall C., Greer P.A., Craig A.W.B. (2013). Calpains promote neutrophil recruitment and bacterial clearance in an acute bacterial peritonitis model. Eur. J. Immunol..

[99] Perez J., Dansou B., Hervé R., Levi C., Tamouza H., Vandermeersch S., Demey-Thomas E., Haymann J.-P., Zafrani L., Klatzmann D. (2016). Calpains Released by T Lymphocytes Cleave TLR2 To Control IL-17 Expression. J. Immunol..

[100] Gößwein S., Lindemann A., Mahajan A., Maueröder C., Martini E., Patankar J., Schett G., Becker C., Wirtz S., Naumann-Bartsch N. (2019). Citrullination Licenses Calpain to Decondense Nuclei in Neutrophil Extracellular Trap Formation. Front. Immunol..

[101] Wang Y., Du F., Hawez A., Mörgelin M., Thorlacius H. (2019). Neutrophil extracellular trap-microparticle complexes trigger neutrophil recruitment via high-mobility group protein 1 (HMGB1)-toll-like receptors (TLR2)/TLR4 signalling. Br. J. Pharmacol..

[102] Chan A.L., Louie S., Leslie K.O., Juarez M.M., Albertson T.E. (2011). Cutting Edge Issues in Goodpasture’s Disease. Clin. Rev. Allergy Immunol..

[103] Mayadas T.N., Rosetti F., Ernandez T., Sethi S. (2010). Neutrophils: Game changers in glomerulonephritis?. Trends Mol. Med..

[104] Lim A.K.H., Tesch G.H. (2012). Inflammation in Diabetic Nephropathy. Mediat. Inflamm..

[105] Martínez-Klimova E., Aparicio-Trejo O.E., Tapia E., Pedraza-Chaverri J. (2019). Unilateral Ureteral Obstruction as a Model to Investigate Fibrosis-Attenuating Treatments. Biomolecules.

[106] Linke A., Tiegs G., Neumann K. (2022). Pathogenic T-Cell Responses in Immune-Mediated Glomerulonephritis. Cells.

[107] Klahr S., Morrissey J. (2002). Obstructive nephropathy and renal fibrosis. Am. J. Physiol. Physiol..

[108] Wu Y.-L., Xie J., An S.-W., Oliver N., Barrezueta N.X., Lin M.-H., Birnbaumer L., Huang C.-L. (2016). Inhibition of TRPC6 channels ameliorates renal fibrosis and contributes to renal protection by soluble klotho. Kidney Int..

[109] Coers W., Huitema S., Van Der Horst M.L., Weening J.J. (1994). Puromycin aminonucleoside and adriamycin disturb cytoskeletal and extracellular matrix protein organization, but not protein synthesis of cultured glomerular epithelial cells. Exp. Nephrol..

[110] Löwenborg E.K.M., Jaremko G., Berg U.B. (2000). Glomerular function and morphology in puromycin aminonucleoside nephropathy in rats. Nephrol. Dial. Transplant..

[111] Wang Z., Wei X., Zhang Y., Ma X., Li B., Zhang S., Du P., Zhang X., Yi F. (2009). NADPH Oxidase-derived ROS Contributes to Upregulation of TRPC6 Expression in Puromycin Aminonucleoside-induced Podocyte Injury. Cell. Physiol. Biochem..

[112] Diaeresisller C.C.M., Wei C., Altintas M.M., Li J., Greka A., Ohse T., Pippin J.W., Rastaldi M.P., Wawersik S., Schiavi S. (2007). Induction of TRPC6 Channel in Acquired Forms of Proteinuric Kidney Disease. J. Am. Soc. Nephrol..

[113] Hall G., Wang L., Spurney R.F. (2019). TRPC Channels in Proteinuric Kidney Diseases. Cells.

[114] Zhang S., Wang H., Liu Y., Yang W., Liu J., Han Y., Liu Y., Liu F., Sun L., Xiao L. (2020). Tacrolimus ameliorates tubulointerstitial inflammation in diabetic nephropathy via inhibiting the NFATc1/TRPC6 pathway. J. Cell. Mol. Med..

[115] Fu Y., Wang C., Zhang D., Xin Y., Li J., Zhang Y., Chu X. (2018). Increased TRPC6 expression is associated with tubular epithelial cell proliferation and inflammation in diabetic nephropathy. Mol. Immunol..

[116] Ma R., Liu L., Jiang W., Yu Y., Song H. (2015). FK506 ameliorates podocyte injury in type 2 diabetic nephropathy by down-regulating TRPC6 and NFAT expression. Int. J. Clin. Exp. Pathol..

[117] Shen B., He Y., Zhou S., Zhao H., Mei M., Wu X. (2016). TRPC6 May Protect Renal Ischemia-Reperfusion Injury Through Inhibiting Necroptosis of Renal Tubular Epithelial Cells. Experiment.

[118] Shen B., Mei M., Pu Y., Zhang H., Liu H., Tang M., Pan Q., He Y., Wu X., Zhao H. (2019). Necrostatin-1 Attenuates Renal Ischemia and Reperfusion Injury via Meditation of HIF-1α/mir-26a/TRPC6/PARP1 Signaling. Mol. Ther.-Nucleic Acids.

[119] Goldwich A., Burkard M., Ölke M., Daniel C., Amann K., Hugo C., Kurts C., Steinkasserer A., Gessner A. (2013). Podocytes Are Nonhematopoietic Professional Antigen-Presenting Cells. J. Am. Soc. Nephrol..

[120] Baker K., Rath T., Pyzik M., Blumberg R.S. (2014). The Role of FcRn in Antigen Presentation. Front. Immunol..

[121] Dylewski J., Tonsawan P., Garcia G., Lewis L., Blaine J. (2020). Podocyte-specific knockout of the neonatal Fc receptor (FcRn) results in differential protection depending on the model of immune-mediated kidney disease. PLoS ONE.

[122] Tonsawan P., Dylewski J., Lewis L., Blaine J. (2019). Knockout of the neonatal Fc receptor in cultured podocytes alters IL-6 signaling and the actin cytoskeleton. Am. J. Physiol. Physiol..

[123] Maschalidi S., Nunes-Hasler P., Nascimento C.R., Sallent I., Lannoy V., Garfa-Traore M., Cagnard N., Sepulveda F.E., Vargas P., Lennon-Duménil A.-M. (2017). UNC93B1 interacts with the calcium sensor STIM1 for efficient antigen cross-presentation in dendritic cells. Nat. Commun..

[124] Nunes-Hasler P., Maschalidi S., Lippens C., Castelbou C., Bouvet S., Guido D., Bermont F., Bassoy E.Y., Page N., Merkler D. (2017). STIM1 promotes migration, phagosomal maturation and antigen cross-presentation in dendritic cells. Nat. Commun..

[125] Heiner I., Eisfeld J., Halaszovich C.R., Wehage E.M., Jüngling E., Zitt C., Lückhoff A. (2003). Expression profile of the transient receptor potential (TRP) family in neutrophil granulocytes: Evidence for currents through long TRP channel 2 induced by ADP-ribose and NAD. Biochem. J..

[126] Kim E.Y., Anderson M., Dryer S.E., Guo H., Wang B., Li H., Ling L., Niu J., Gu Y., Ilatovskaya D.V. (2012). Insulin increases surface expression of TRPC6 channels in podocytes: Role of NADPH oxidases and reactive oxygen species. Am. J. Physiol. Physiol..

[127] Johnson R.J., Couser W.G., Chi E.Y., Adler S., Klebanoff S.J. (1987). New mechanism for glomerular injury. Myeloperoxidase-hydrogen peroxide-halide system. J. Clin. Investig..

[128] Li J.Z., Sharma R., Dileepan K.N., Savin V.J. (1994). Polymorphonuclear leukocytes increase glomerular albumin permeability via hypohalous acid. Kidney Int..

[129] Ortiz-Muñoz G., Yu M.A., Lefrançais E., Mallavia B., Valet C., Tian J.J., Ranucci S., Wang K.M., Liu Z., Kwaan N. (2020). Cystic fibrosis transmembrane conductance regulator dysfunction in platelets drives lung hyperinflammation. J. Clin. Investig..

[130] Scherlinger M., Guillotin V., Truchetet M.-E., Contin-Bordes C., Sisirak V., Duffau P., Lazaro E., Richez C., Blanco P. (2018). Systemic lupus erythematosus and systemic sclerosis: All roads lead to platelets. Autoimmun. Rev..

[131] Joseph J.E., Harrison P., Mackie I.J., Isenberg D.A., Machin S.J. (2001). Increased circulating platelet–leucocyte complexes and platelet activation in patients with antiphospholipid syndrome, systemic lupus erythematosus and rheumatoid arthritis. Br. J. Haematol..

[132] Pieterse E., Rother N., Garsen M., Hofstra J.M., Satchell S.C., Hoffmann M., Loeven M.A., Knaapen H.K., van der Heijden O.W., Berden J.H. (2017). Neutrophil Extracellular Traps Drive Endothelial-to-Mesenchymal Transition. Arter. Thromb. Vasc. Biol..

[133] Vemana H.P., Karim Z.A., Conlon C., Khasawneh F.T. (2015). A Critical Role for the Transient Receptor Potential Channel Type 6 in Human Platelet Activation. PLoS ONE.

[134] Lopez E., Bermejo N., Berna-Erro A., Alonso N., Salido G., Redondo P., Rosado J. (2015). Relationship between calcium mobilization and platelet α- and δ-granule secretion. A role for TRPC6 in thrombin-evoked δ-granule exocytosis. Arch. Biochem. Biophys..

[135] Espinosa E.V.P., Lin O.A., Karim Z.A., Alshbool F.Z., Khasawneh F.T. (2019). Mouse transient receptor potential channel type 6 selectively regulates agonist-induced platelet function. Biochem. Biophys. Rep..

[136] Espinosa E.V.P., Murad J.P., Ting H.J., Khasawneh F.T. (2012). Mouse transient receptor potential channel 6: Role in hemostasis and thrombogenesis. Biochem. Biophys. Res. Commun..

[137] Ramanathan G., Mannhalter C. (2015). Increased expression of transient receptor potential canonical 6 (TRPC6) in differentiating human megakaryocytes. Cell Biol. Int..

[138] Tesch G.H. (2017). Diabetic nephropathy–is this an immune disorder?. Clin. Sci..

[139] Chaudhuri P., Putta P., Rosenbaum M.A., Graham L.M. (2023). p38 MAPK activation and STIM1-Orai3 association mediate TRPC6 externalization. Am. J. Physiol. Physiol..

[140] Shen B., Zhou S., He Y., Zhao H., Mei M., Wu X. (2013). Revealing the Underlying Mechanism of Ischemia Reperfusion Injury Using Bioinformatics Approach. Kidney Blood Press. Res..

[141] Zhao B., Yang H., Zhang R., Sun H., Liao C., Xu J., Meng K., Jiao J. (2015). The role of TRPC6 in oxidative stress-induced podocyte ischemic injury. Biochem. Biophys. Res. Commun..

[142] Cobo G., Lindholm B., Stenvinkel P. (2018). Chronic inflammation in end-stage renal disease and dialysis. Nephrol. Dial. Transplant..

[143] Naicker S.D., Cormican S., Griffin T.P., Maretto S., Martin W.P., Ferguson J.P., Cotter D., Connaughton E.P., Dennedy M.C., Griffin M.D. (2018). Chronic Kidney Disease Severity Is Associated With Selective Expansion of a Distinctive Intermediate Monocyte Subpopulation. Front. Immunol..

[144] Chen C.-M., Juan S.-H., Chou H.-C. (2018). Hyperglycemia activates the renin-angiotensin system and induces epithelial-mesenchymal transition in streptozotocin-induced diabetic kidneys. J. Renin.-Angiotensin.-Aldosterone. Syst..

[145] Ilatovskaya D.V., Levchenko V., Lowing A., Shuyskiy L.S., Palygin O., Staruschenko A. (2015). Podocyte injury in diabetic nephropathy: Implications of angiotensin II – dependent activation of TRPC channels. Sci. Rep..

[146] Liu B.-C., Song X., Lu X.-Y., Li D.T., Eaton D.C., Shen B.-Z., Li X.-Q., Ma H.-P. (2013). High glucose induces podocyte apoptosis by stimulating TRPC6 via elevation of reactive oxygen species. Biochim. Et Biophys. Acta (BBA)-Mol. Cell Res..

[147] Ilatovskaya D.V., Blass G., Palygin O., Levchenko V., Pavlov T.S., Grzybowski M.N., Winsor K., Shuyskiy L.S., Geurts A.M., Cowley A.W. (2018). A NOX4/TRPC6 Pathway in Podocyte Calcium Regulation and Renal Damage in Diabetic Kidney Disease. J. Am. Soc. Nephrol..

[148] Lech M., Anders H.-J. (2013). The Pathogenesis of Lupus Nephritis. J. Am. Soc. Nephrol..

[149] Clark M.R., Trotter K., Chang A. (2015). The Pathogenesis and Therapeutic Implications of Tubulointerstitial Inflammation in Human Lupus Nephritis. Semin. Nephrol..

[150] Ramirez G.A., Coletto L.A., Bozzolo E.P., Citterio L., Carpini S.D., Zagato L., Rovere-Querini P., Lanzani C., Manunta P., Manfredi A.A. (2018). The TRPC6 intronic polymorphism, associated with the risk of neurological disorders in systemic lupus erythematous, influences immune cell function. J. Neuroimmunol..

[151] Dos Santos M., Bringhenti R.N., Rodrigues P.G., Nascimento J.F.D., Pereira S.V., Zancan R., A Monticielo O., A Gasparin A., De Castro W.P., Veronese F.V. (2015). Podocyte-associated mRNA profiles in kidney tissue and in urine of patients with active lupus nephritis. Int. J. Clin. Exp. Pathol..

[152] Elloumi N., Ben Mansour R., Marzouk S., Mseddi M., Fakhfakh R., Gargouri B., Masmoudi H., Lassoued S. (2017). Differential reactive oxygen species production of neutrophils and their oxidative damage in patients with active and inactive systemic lupus erythematosus. Immunol. Lett..

[153] Lindberg U., Svensson L., Hellmark T., Segelmark M., Shannon O. (2018). Increased platelet activation occurs in cystic fibrosis patients and correlates to clinical status. Thromb. Res..

[154] Martínez-Alemán S.R., Campos-García L., Palma-Nicolas J.P., Hernández-Bello R., González G.M., Sánchez-González A. (2017). Understanding the Entanglement: Neutrophil Extracellular Traps (NETs) in Cystic Fibrosis. Front. Cell. Infect. Microbiol..

[155] Pieterse E., Rother N., Yanginlar C., Hilbrands L.B., van der Vlag J. (2016). Neutrophils Discriminate between Lipopolysaccharides of Different Bacterial Sources and Selectively Release Neutrophil Extracellular Traps. Front. Immunol..

[156] Clark S.R., Ma A.C., Tavener S.A., McDonald B., Goodarzi Z., Kelly M.M., Patel K.D., Chakrabarti S., McAvoy E., Sinclair G.D. (2007). Platelet TLR4 activates neutrophil extracellular traps to ensnare bacteria in septic blood. Nat. Med..

[157] Kistler A.D., Singh G., Altintas M.M., Yu H., Fernandez I.C., Gu C., Wilson C., Srivastava S.K., Dietrich A., Walz K. (2013). Transient Receptor Potential Channel 6 (TRPC6) Protects Podocytes during Complement-mediated Glomerular Disease. J. Biol. Chem..

[158] Wang L., Chang J.-H., Buckley A.F., Spurney R.F. (2019). Knockout of TRPC6 promotes insulin resistance and exacerbates glomerular injury in Akita mice. Kidney Int..

[159] Block K., Eid A., Griendling K.K., Lee D.-Y., Wittrant Y., Gorin Y. (2008). Nox4 NAD(P)H Oxidase Mediates Src-dependent Tyrosine Phosphorylation of PDK-1 in Response to Angiotensin II. J. Biol. Chem..

[160] Eid A.A., Gorin Y., Fagg B.M., Maalouf R., Barnes J.L., Block K., Abboud H.E. (2009). Mechanisms of Podocyte Injury in Diabetes. Diabetes.

[161] Mapanga R.F., Essop M.F., Das R., Xu S., Quan X., Nguyen T.T., Kong I.D., Chung C.H., Lee E.Y., Cha S.-K. (2006). Mesangial cell NADPH oxidase upregulation in high glucose is protein kinase C dependent and required for collagen IV expression. Am. J. Physiol. Physiol..

[162] Rajaram R.D., Dissard R., Faivre A., Ino F., Delitsikou V., Jaquet V., Cagarelli T., Lindenmeyer M., Jansen-Duerr P., Cohen C. (2019). Tubular NOX4 expression decreases in chronic kidney disease but does not modify fibrosis evolution. Redox Biol..

[163] A Study to Test BI 764198 in People with a Type of Kidney Disease Called Primary Focal Segmental Glomerulosclerosis. https://classic.clinicaltrials.gov/ct2/show/NCT05213624.

